# Between-breed variations in resistance/resilience to gastrointestinal nematodes among indigenous goat breeds in Uganda

**DOI:** 10.1007/s11250-017-1390-9

**Published:** 2017-09-13

**Authors:** R. B. Onzima, R. Mukiibi, A. Ampaire, K. K. Benda, E. Kanis

**Affiliations:** 1 Wageningen University and Research - Animal Breeding and Genomics Centre, P.O. Box 338, 6700AH Wageningen, Netherlands; 2 National Agricultural Research Organization (NARO), P.O. Box 295, Entebbe, Uganda; 3 Department of Agriculture, Food and Nutritional Sciences (AFNS), Faculty of Agriculture, Life and Environmental Sciences University of Alberta, 1416 College Plaza, Edmonton, Alberta T6G 2C8 Canada

**Keywords:** *Haemonchus contortus*, Small East African, Mubende, Kigezi, Breeding programs

## Abstract

Gastrointestinal nematodes (GINs), *Haemonchus contortus*, are a major health problem in goat production. Resistance to *H. contortus*, the most prevalent GIN in Uganda, was studied among three indigenous goat breeds to assess their differences. Twelve male goats of each breed approximately 7 months old of small East African (SEA), Mubende, and Kigezi goats from smallholder farmers in Arua, Mubende, and Kabale were assembled for the study. At the station, they were dewormed with a combination therapy of the broad-spectrum dewormers closantel and albendazole to free the goats of gastrointestinal parasites. During experimentation, the goats were kept indoors and ad libitum fed on clean banana peels and napier grass. On attainment of zero-worm-egg status, the goats were artificially infected with 18,000 third-stage (L3) larvae of *H. contortus* prepared according to Baermann’s procedure. Data were collected on fecal egg count (FEC), packed cell volume (PCV), and body weight (BW) on a 2-week basis until 12 weeks post infection and carcass weight and total worm count (WC) in the abomasum at termination of the experiment. The data on FEC, PCV, and BW were subjected to repeated-measure analysis of variance and the others by one-way analysis of variance. FEC between breeds was only significantly different at 12 weeks post infection (*p* = 0.04). Generally, higher FEC was recorded in Kigezi compared to SEA and Mubende goats. Carcass weight was significantly different among breeds (*p* < 0.05), with Mubende having the highest carcass weight, followed by Kigezi and SEA. PCV and daily weight gains were significantly different between breeds (*p* < 0.05). WC was not significantly different between the breeds. FEC and PCV were weakly significant at later stages of the experiment with higher parasite burden suggesting potential variation in resistance to *H. contortus*. These differences could be exploited in designing breeding programs with disease resistance in indigenous goat breeds.

## Introduction

The gastrointestinal nematode (GIN) *Haemonchus contortus* is a major health problem, causing immense economic losses in goat production (Baker et al. 1998; Bambou et al. 2013; Campos et al. 2009; Mandonnet et al. 2001). In Australia, the annual losses due to GIN is estimated at over AUD 400 million (McLeod 1995; Sacket et al. 2006). It is similarly estimated that the costs of diseases generally may be as high as 35–50% of turnover within the livestock sector in developing countries (Bishop 2012). In French West Indies, losses in goat farm profit of up to 81% have been reported (Gunia et al. 2013). The losses have mainly been attributed to significant morbidity and mortality in small ruminants.

Helminthosis (diseases including those caused by nematode parasites) is the most important livestock disease in most tropical countries (Khan et al. 2010; Lapenga et al. 2009; Perry et al. 2002; Vatta and Lindberg 2006). The most prevalent of GINs in Uganda is *H. contortus* (Nsereko et al. 2015). GIN particularly *H. contortus* has a high fecundity and is a debilitating blood-sucking parasite in the abomasum causing significant production losses through severe chronic anemia, anorexia, loss of condition, and eventual death of the affected animals (Notter et al. 2003).

Options for control of GINs include vaccination, chemotherapy, improved management, and utilization of host genetic variation. The control of gastrointestinal parasites in many livestock production systems has been mainly through the use of anthelminthics. However, the development of resistance to major anthelminthics (Kaplan 2004; Zajac and Gipson 2000) and increasing consumer health concerns over occurrence of drug residues in food have further complicated control of nematodes. The decreasing efficacy of anthelminthics coupled with the desire for less chemical use in production systems has stimulated search for alternatives for sustainable control of parasites. Genetic resistance against GINs is well described within and between sheep breeds and to a lesser extent in some goat breeds (Bishop and Stear 2003; Fakae et al. 2004; Gruner et al. 2004; Mandonnet et al. 2001). The use of relatively resistant animals improves growth, and there is less contamination of pastures, thus minimizing the need for anthelmintics and the development of anthelmintic resistance (Matika et al. 2003).

Resistance in animals is the ability of breeds to suppress establishment and subsequent development of infection (Albers et al. 1987), while resilience or tolerance refers to the ability of the host/animal to survive and remain productive despite parasite challenge (Bishop 2012). Resistance to GI parasites can be exhibited by a lower fecal egg count (FEC), while resilience is commonly measured with the packed cell volume (PCV) (Bisset and Morris 1996) and increase in body weight (BW) in growing animals. The PCV measures the volume of circulating erythrocytes in the blood compared to the whole blood, making it a suitable cutoff point for anemia in animals. There is consensus that a ruminant with a PCV of 20–26% may have a mild anemia (Tvedten 2010), and the normal range in goats is estimated to be from 22 to 38% (Byers and Kramer 2010). Thus, any animal with a PCV below 20% can be considered to be anemic and not resilient to the GIN.

Goats are markedly susceptible to GINs. However, there are breeds and individuals which are genetically resistant to nematodes (Behnke et al. 2006; Pralomkarn et al. 1997). These breeds and animals show a low FEC and parasite survival (worm burden). Worm burden is a measure of the number of adult worms in an individual host. A positive correlation may exist between fecal egg counts and worm counts/burden (Cabaret and Gasnier 1994; Cabaret et al. 1998). Therefore, FEC may be used as an indicator for worm burden. However, there is skepticism over this approach in that the worms are not randomly distributed in the hosts. Low levels of FEC and worm burdens are closely associated and are good indicators of resistant animals. It may therefore be possible to select for improved resistance to nematodes in goats (Mandonnet et al. 2001, 2006).

Studies with sheep show a marked susceptibility of male lambs to GINs compared to female lambs, arising from both natural and experimental infections. This may be attributed to sex steroids (androgens vs. estrogens) which control several aspects of host immunity (Klein 2000a, b). Therefore, the use of male animals is envisaged to give a large (and variable) response in challenge studies.

Evidence for variation in resistance to GIN, particularly *H. contortus*, between breeds has been extensively documented in sheep (Alba-Hurtado et al. 2010; Amarante et al. 2004; Getachew et al. 2015; González et al. 2011; Haile et al. 2002). A few studies confirm the existence of resistance in indigenous goats in Africa, for example, the West African dwarf (WAD) goats (Chiejina and Behnke 2011) and Kenyan small East African (SEA) goats (Baker et al. 1998). However, there is no known scientific literature about the variation in resistance/resilience to GIN among the indigenous goat breeds of Uganda.

The objective of this study was therefore to evaluate the variation in resistance/resilience in three indigenous goat breeds of Uganda when artificially infected with the GIN *H. contortus*.

## Material and methods

### Experimental animals and management

An on-station challenge experiment was conducted at the Kachwekano Zonal Agricultural Research and Development Institute (KAZARDI) in Kabale (1.33° S, 30.00° E, 1864 m above sea level (masl)). Twelve male kids each from the three main indigenous goat breeds in Uganda were purchased from smallholder farmers in Kabale (1.33° S, 30.00° E, 1864 masl), Mubende (0.59° N, 31.36° E, and 1324 masl), and Arua (3.03° N, 30.91° E, and 1157 masl), where Kigezi (KIG), Mubende (MUB), and small East African (SEA) goats, respectively, were predominantly found. Only male kids were used to eliminate a possible sex effect, and moreover, male goats were easily accessible across all production systems in Uganda. The goats used in this experiment were approximately 7 months of age at the time of purchase and selected from different parental lines in widely distributed locations to ensure that they were not related to each other. Goats at this age were commonly believed to be immunologically immature (Hoste et al. 2010), and the males are generally considered more susceptible than females and were therefore suitable for the study. The management at the farms was similar where the kids were raised together with the does and grazed together on natural grass. While on station, the animals were subjected to the same management (feeding and housing) prior to experimentation. They were initially drenched with a combination therapy of nilzan plus albendazole 10%, followed by a closantel treatment where the parasite was not cleared by nilzan plus albendazole combination therapy. The goats were then allowed to adjust to the prevailing husbandry conditions for a period of 4–6 weeks. Briefly, on arrival at the station, the animals were kept in isolation and sprayed against ectoparasites, particularly ticks, and treated against enterotoxemia according to approved standard procedures. During the quarantine period, their health was closely monitored and any ailment that may have an adverse effect on the goats was treated promptly. To avoid secondary infection, the experiment was performed under a cut-and-carry system of feeding. The goats were fed on clean banana peels and napier grass (*Pennisetum purpureum*) as the basal diet and were supplemented with legumes, mainly lablab (*Lablab purpureus*) and calliandra (*Calliandra calothyrsus*) forage, throughout the experimental period. The fodder was obtained from established pastures not previously grazed by animals but mainly used for cut-and-carry feeding. Mineral blocks rich in essential minerals were availed in each pen to meet their mineral requirements. Water was provided to the goats ad libitum, and each pen with goats was kept clean at all times to minimize secondary infection. Experimental management and data collection were performed using approved ethical standards of the Uganda National Council of Science and Technology (UNCST) (SBLS/REC/15/131).

### Worm cultures and inoculation

**Fig. 1:**
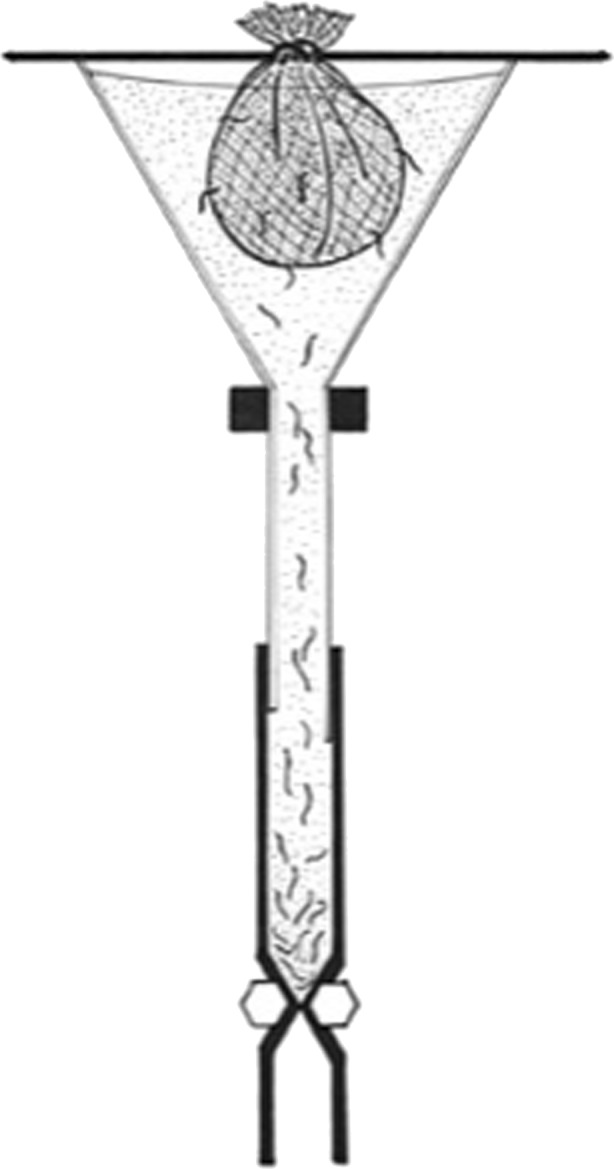
Baermann apparatus (*Adapted from* Hansen and Perry 1994)

**Plate 1:**
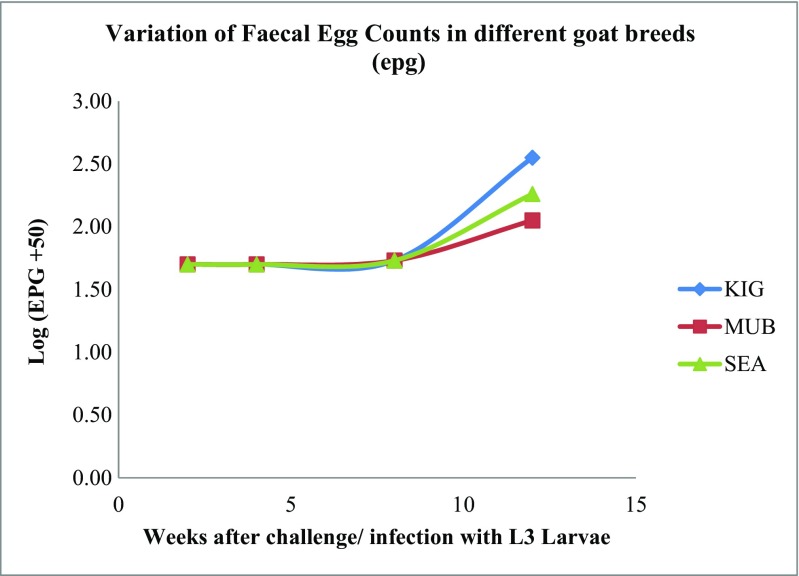
*Haemonchus contortus* larvae as seen under the microscope (× 100)

Grazing goats are usually infected by third-stage larvae (L3) of *H. contortus* (Hoste et al. 2010)*.* However, worm infections tend to occur as mixed proportions of helminths. To obtain pure cultures of *H. contortus*, goats were sampled from several herds and two goats with over 90% infection by *H. contortus* were recruited as source for developing pure *H. contortus* cultures. Briefly, fecal samples were obtained from the donors and female fully engorged *Haemochus contortus* was isolated and incubated in sterile fecal material at a temperature of 27 °C for 14 days before harvesting the L3 larvae using Baermann’s technique (Hansen and Perry 1994). The technique works on the principle of active migration of the larvae, allowing them to move into the water and sink into the bottom of the Baermann’s apparatus to be collected, while the fecal particles remain suspended in water (Fig*.* 1). The larval cultures (Plate 1) were stored at 10 °C in the fridge until used. The viability of the larvae was ascertained before administering to the goats to ensure that sufficient living larvae were fed to the experimental goats. Ten to fourteen days prior to artificially infecting the goats with L3 larvae, a thorough fecal examination is undertaken to ensure that the experimental goats were free of nematode eggs.

Twelve goats of each breed were assigned to three different breed groups (KIG, MUB, SEA) in different pens. Prior to infection of the goats, the viability of the L3 larvae was ascertained microscopically and the number of living larvae was counted for every oral admintering. Eighteen thousand infective L3 larvae were administered to the goats orally using a syringe.

### Data

Data collection commenced from week 3 post-infection and continued for 10 weeks. During experimentation, FEC, PCV, and BW for each animal were measured once every week. For FEC, fresh fecal samples were taken thrice weekly in the morning hours, directly from the rectum of the experimental animals, by rectal palpitation by a trained and skilled technician to minimize soft tissue damage. During the experimentation period, the physiological status of animals was closely observed.

#### Parasitological data

FEC was determined for each individual feces sample and averaged per animal per week. The data were expressed as average number of eggs per gram feces (epg) (Fakae et al. 1999). To obtain FEC, 20 g of fresh fecal samples were collected directly from the rectum of each animal and processed individually with modified McMaster technique (MAFF 1986) using standard operating procedures. With the procedure, 3 g of feces was put into a container and a saturated NaCl solution was added. The resultant suspension was thoroughly homogenized and strained through a sieve to remove large debris. The strained fecal samples were then examined for worm eggs on a McMaster slide under a light microscope using a × 100 magnification (Cringoli et al. 2004). Meanwhile, BW was determined on a weekly basis using a sensitive scale.

At the termination of the experiment (day 84), all animals were slaughtered and WC was determined by the number of adult worms recovered from the abomasum (Hoste et al. 2010). Briefly, the abomasum was separated and opened and contents were emptied into a volumetric flask. The lining mucosa was then thoroughly washed with warm 0.9% NaCl to detach any adherent nematodes and added to the respective abomasal contents. An aliquot of the washings and contents was assessed for nematode worms to establish the WC.

#### Hematology

PCV was measured using a hematocrit reader according to the procedure of (Dacie and Lewis 1995). PCV measurements were taken at the start of the experiment (day 0) and subsequently on a weekly basis up to week 12 (day 84) post-infection. Blood samples for PCV determination were collected from the jugular vein into an ethylenediamine tetra-acetic acid (EDTA)-coated vacutainer tube. The freshly collected blood samples were subjected to hematocrit analysis to obtain the PCV values.

### Data analysis

Data on the effect of breed on CW, FEC, WC, PCV, and BW at different time periods were tested using linear mixed model implemented by the R package *nlme* (R Core Team 2016) with goat identification(goat-id) as random factor accounting for spatial correlation of the measurements in the same goat. Data for FEC and WC were first log-transformed to normalize the data before statistical analysis. The model used included fixed effects of breed, week of measurement, breed-week interactions, and a random (repeated) effect of the goat-id.

1 $$ {Y}_{ijk}=\mu +{\tau}_i+{\beta}_j+{\tau}_i{\beta}_j+{\varepsilon}_{ijk} $$

where *Y*_*ijk*_ is the measurement (FEC, BW, or PCV) on the *k*th individual within the *i*th breed and the *j*th week, *μ* is the overall mean, *τ*_*i*_ is the effect of breed *i*, *β*_*j*_ is the effect of week *j*, *τ*_*i*_ *β*_*j*_ is effect of the interaction between the *i*th breed and the *j*th week, and *ε*_*ijk*_ is the error term. One-way analysis of variance (ANOVA) in R was used to test for breed differences in carcass weight (CW) and WC.

2 $$ {Y}_{ij}=\mu +{\tau}_i+{\varepsilon}_{ij} $$

where *Y*_*ij*_ is the individual measure of CW or WC for the *j*th goat from the *i*th breed and *τ*_*i*_ is the effect of the *i*th breed.

## Results

The overall variation in FEC (logEPG), PCV, and WC between the breeds is represented in Table 1. Log-transformed eggs per gram (LEPG) were not significantly different between the breeds, although the numerical values were higher in KIG, followed by SEA and the lowest in MUB. The PCV in SEA was significantly lower compared to KIG and MUB. WC was not significantly different between breeds (*P* > 0.05). However, there were a higher number of adult worms in KIG compared to MUB and SEA goats.

**Table 1:** Least square means ± standard error of log FEC, packed cell volume, and total worm count of *Haemonchus contortus-*infected goats

Breed	LFEC(logEPG)	PCV	Total worm count
Kigezi	1.917a ± 0.040	29.682a ± 1.096	25.364a ± 6.052
Mubende	1.792a ± 0.042	30.350a ± 1.150	16.800a ± 6.348
Small East African (SEA)	1.847a ± 0.044	25.750b ± 1.212	16.000a ± 6.691

Means within the same column with differing lowercase letters differ significantly (*p* < 0.05)

**Fig. 2:**
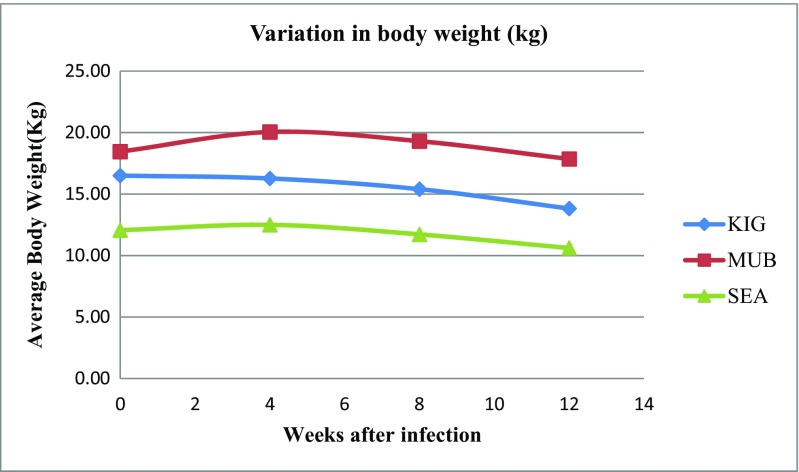
Change in FEC over time in Kigezi, Mubende, and small East African (SEA) goats

The variation in the fecal egg counts in the different breeds during the period of challenge is shown in Table 2. FECs were similar among the three indigenous breeds. Although the FECs differed numerically, they were not statistically significant (*P* > 0.05) at the initial stages of the experiment. However, at week 12, FEC was marginally significant (*P* = 0.04) with the highest value recorded in KIG goats. The differences between SEA and MUB goats remained similar statistically throughout the experiment. FEC data was recorded after 8 weeks post infection (PI) as shown in Fig. 2 *.*

**Table 2:** Log-transformed fecal egg counts (LEPG) in three different indigenous goat breeds

Weeks (post infection)	Log fecal egg counts (mean ± SD)		
	Kigezi	Mubende	Small East African (SEA)
2	1.699a ± 0.000	1.699a ± 0.000±	1.699a ± 0.000
4	1.699a ± 0.000	1.699a ± 0.000	1.699a ± .000
8	1.726a ± 0.908	1.729a ± 0.952	1.732a ± 0.100
12	2.545a ± 0.522	2.054bc ± 0.472	2.256ac ± 0.546

Means within the same row with differing lowercase letters differ significantly (*p* < 0.05)

**Fig. 3:**
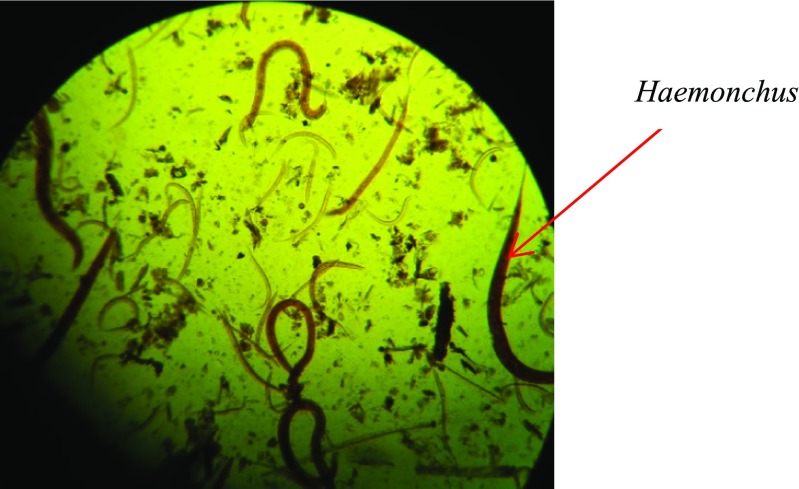
Variation in average live body weight of breeds post infection (PI) with L3 larvae of *Haemochus contortus*

The live BW, carcass weight, and average daily gains are shown in Table 3. The average live body weight and carcass weights were significantly different among the breeds (*P* < 0.05). The highest body weight was found in Mubende, followed by Kigezi and SEA, respectively. It was generally higher in Mubende. There was a general reduction in average body weight PI with L3 larvae of *H. contortus* (Fig*.* 3). Daily weight gain (DWG) was significantly different (*P* < 0.05) in MUB compared to SEA and KIG. The highest weight loss was recorded in KIG followed by SEA with MUB, respectively.

**Table 3:** Least square means and standard error for live body weight, carcass weight, and daily weight gain

Breed	Body weight (kg)	Carcass weight (kg)	Daily weight gain
Kigezi	14.614a ± 0.565	5.818a ± 0.302	− 0.030a ± 0.005
Mubende	18.575b ± 0.593	7.650b ± 0.317	− 0.007b ± 0.005
Small East African (SEA)	11.167c ± 0.625	4.389c ± 0.334	− 0.016a ± 0.006

Means within the same column with differing lowercase letters differ significantly (*p* < 0.05)

## Discussion

Difference between breeds to resistance to gastrointestinal parasites is always limited by the small numbers of experimental animals per breed or genotypes being compared. This often affects the ability to draw meaningful conclusions from such studies (Marume et al. 2011). In the present study, we did not find a significant difference in FEC between the breeds, although PCV in SEA was significantly lower than for KIG and MUB (Table 1). The findings in the current study agree with the findings of Costa et al. (2000) working with three goat breeds in Brazil, who found similar FEC but significantly different PCV values between breeds. PCV is a good indicator for resilience when the GIN is a blood-sucking parasite like *H. contortus* (Baker et al. 2001). In this regard, MUB with the highest PCV showed a relatively high degree of resilience to GIN particularly *H. contortus* and the lowest resilience was found with SEA which showed the lowest PCV. Overly, PCV across the breeds was within the 22–38% range recognized as the normal range in goats by Byers and Kramer (2010). This may have been due to the low levels of infection recorded as the parasite established itself in the host as indicated by the gradual rise in FEC (Table 2). In the present study, FEC was not significant; however, it is noteworthy here that there is a close correlation between anemia and PCV in *H. contortus* infection in both sheep (Albers et al. 1987) and goats (Baker et al. 1998). We conclude from this finding that MUB may probably present as the most resistant of the three breeds under study. Further, a plot of LEPG over time (Fig*.* 2), shows invariably lower values in MUB compared to KIG and SEA. The differences in FEC and PCV could be attributed to breed genetic differences. Genetic differences in resistance/resilience in goats have been previously reported in West African dwarf goats (Chiejina and Behnke 2011) and SEA goats in Kenya by Baker and Gray (2004) and Baker et al. (1998). Similarly, the difference reported in this study may be under genetic control, thus the possibility of including resistance traits in breed improvement programs for indigenous goats.

Body and carcass weights were all significantly different from each other, and there was a general loss in weight experienced with all the breeds (Fig. 3). However, the daily body weight loss found in MUB was significantly lower (*P* < 0.05) than in SEA and KIG. This implies that MUB was able to maintain a relatively stable weight despite the worm challenge as compared to KIG and SEA. The initial loss in weight during the acclimatization could be attributed to change in feeding system; however, in the long run, other factors may have possibly come into play including the genetic differences.

## Conclusions and recommendations

There was a significant variation in daily weight gain between the breeds suggesting some variation in the effect of the parasite on the different goat breeds.

FEC and PCV were weakly significant at the later stage of the experiment when the parasite burden was increased suggesting variation in a difference in genetic predisposition which could be exploited.

There is a need for long-term study with more recorded individuals to validate the findings from this experiment.

Development of selection population with genomic selection in the long run could become a useful tool in goat breed improvement programs in the tropics.
